# Sexually differential tolerance to water deficiency of *Salix paraplesia*—A female‐biased alpine willow

**DOI:** 10.1002/ece3.5175

**Published:** 2019-07-17

**Authors:** Jun Liao, Haifeng Song, Duoteng Tang, Sheng Zhang

**Affiliations:** ^1^ Key Laboratory of Mountain Surface Processes and Ecological Regulation, Institute of Mountain Hazards and Environment Chinese Academy of Sciences Chengdu China; ^2^ University of Chinese Academy of Sciences Beijing China; ^3^ Key Laboratory of Bio‐Resource and Eco‐Environment of Ministry of Education, College of Life Sciences Sichuan University Chengdu China

**Keywords:** alpine, dioecy, drought, sex ratio, willow

## Abstract

Salicaceae plants are dioecious woody plants. Previous studies have shown that male individuals are more tolerant to water deficiency than females for male‐biased poplars. However, *Salix paraplesia* is a female‐biased species in nature. It is still unknown whether female willows are more tolerant to drought stress than males. To better understand the sexually different tolerance to water deficiency in willows, a greenhouse experiment combined with a field investigation was conducted, and physiological traits were tested in male and female *S. paraplesia* under a drought‐stressed condition (50% of soil water capacity). Our field investigation showed that *S. paraplesia* was a species with female‐biased sex ratio along altitude gradients (2,400 m, 2,600 m and 2,800 m) in their natural habitats. Our results showed that the height growth, biomass accumulation, total chlorophyll pigment content (*TChl*), and the net photosynthetic rate were higher in female willows than in males at the low and middle altitudes (2,400 m and 2,600 m) rather than at a high altitude (2,800 m) under well‐watered conditions. Under drought‐stressed conditions, the growth, biomass, and photosynthesis were greatly inhibited in both sexes, while females showed higher biomass and *TChl* content and suffered less negative effects than did males. Particularly, females that originated from a high altitude showed lower leaf relative electrolyte leakage, malondialdehyde content, and less disorder of chloroplast ultrastructures but a higher peroxidase activity (POD) than that of males. Therefore, *S. paraplesia* females exhibited a better drought tolerance and self‐protective ability than males from high altitude. There is a reason to speculate that the population structure of *S. paraplesia* at a high altitude would be likely to further female biases with the increased drought intensity in the alpine regions.

## 1 INTRODUCTION

Dioecious plants play key roles in maintaining the stability of structure and function in terrestrial ecosystems (Renner, 2014; Renner & Ricklefs, 1995). Under equal and natural environments, a dioecious plant population should be balanced with a 1:1 sex ratio (Fisher, 1930). The theory of ecological causation postulated that male and female plants evolved different secondary characteristics, resulting from different ecological niches (Tognetti, 2012). However, environmental factors can skew the sex ratio by changing morphological and physiological performances. Water is one of the most commonly limited factors in terrestrial forests, and with global warming, the intensity of water deficit will be more serious in the alpine regions (Öztürk, Hakeem, Faridah‐Hanum, & Efe, 2015; Trenberth et al., 2014). Drought with increased elevation influences the sex frequency, fertility patterns, and population dynamics, further leading to a decrease in female bias in *Valeriana edulis* (Petry et al., 2016). Drought can affect not only the growth of individual plants but also the stability of the population structure and even the sex ratio of dioecious plants. Illuminating the sex‐related physiological responses to water limitation is important to predict the population structure and scale of dioecious plants in the future (Tognetti, 2012).

Previous investigations on *Populus cathayana*, a male‐biased alpine tree, have shown that male individuals are more tolerant to drought stress than females because of their higher values of total chlorophyll concentration, net photosynthetic rate, activities of superoxide dismutase, peroxidase, and less negative effects on cellular membranes and chloroplasts (Chen, Duan, Wang, Korpelainen, & Li, 2014; He et al., 2017; Xu, Peng, Wu, Korpelainen, & Li, 2008; Xu, Yang, et al., 2008; Zhang et al., 2010, Zhang, Jiang, Zhao, Korpelainen, & Li, 2014). However, in the genus *Salix*, female individuals occurred more frequently than males in their natural habitats (Dawson & Bliss, 1989; Dudley, 2006; Hughes et al., 2010; Ueno, Suyama, & Seiwa, 2007). It is still unknown whether female willows are more tolerant to drought stress than males. In many alpine plants, most cases showed that female individuals were more tolerant to water limitation than males through a compensatory mechanism, which females exhibited a higher photosynthetic rate or present a higher investment in the formation of symbiosis through mycorrhizal fungi as a mechanism to increase their uptake of soil nutrients to compensate their higher reproductive investment (Álvarez‐Cansino, Zunzunegui, Díaz‐Barradas, & Esquivias, 2010; Obeso, 2002; Rakocevic, Medrado, Martim, & Assad, 2009; Vega‐Frutis, Varga, & Kytöviita, 2013; Wu et al., 2018). For example, *Salix glauca* females showed better growth advantages than males under well‐watered conditions; however, water deficiency decreased the photosynthetic capacity and leaf water potential to a greater extent in females than in males (Dudley, 2006; Dudley & Galen, 2007). Due to a wide genetic diversity in the natural populations of *Salix*, it is impossible to obtain a common conclusion on the sexually differential responses to water limitation. Therefore, more willow species should be investigated.

*Salix paraplesia* is dioecious woody tree or shrub, and is widely distributed in alpine areas at the eastern edge of the Tibetan Plateau (Jiang, Zhang, Lei, Xu, & Zhang, 2016). *Salix paraplesia* is as pioneer tree species that is used for the conservation of soil and water in the high‐altitude areas of China. To examine whether there is a sexually differential tolerance to water limitation for *S. paraplesia*, we conducted a field investigation combined with a greenhouse experiment in their natural habitats. We will answer the following questions: (a) Are *S. paraplesia* females more tolerant to drought stress than males? (b) Are there sex‐related variations in altitude and the interaction response to drought stress?

## 2 MATERIALS AND METHODS

### 2.1 Field investigation of the sex ratio

**Figure 1:**
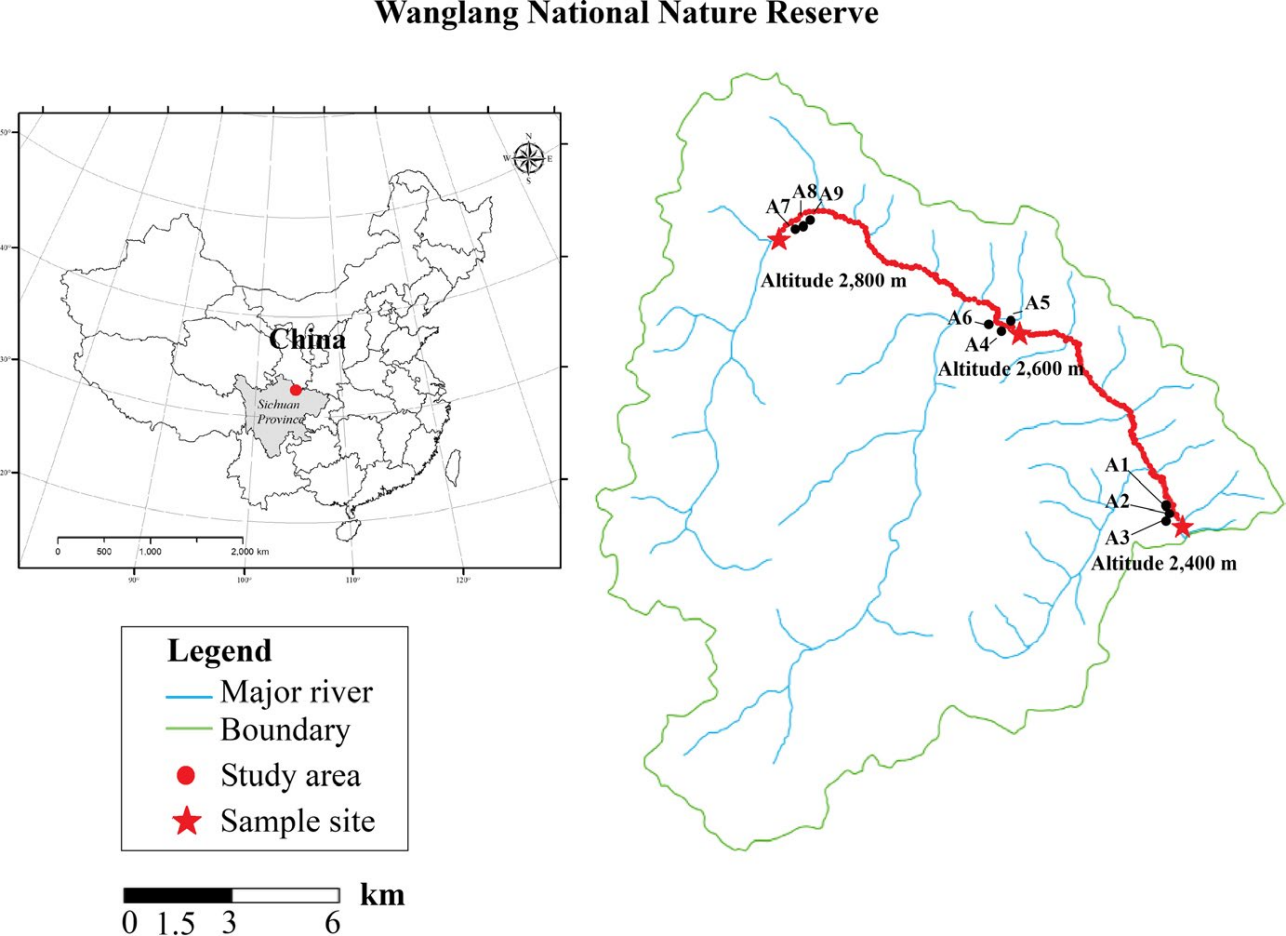
Schematic diagram of regional location and study sites (solid black dots) in the field investigation on sex ratio along altitude gradients (2,400 m, 2,600 m and 2,800 m)

**Figure 2:**
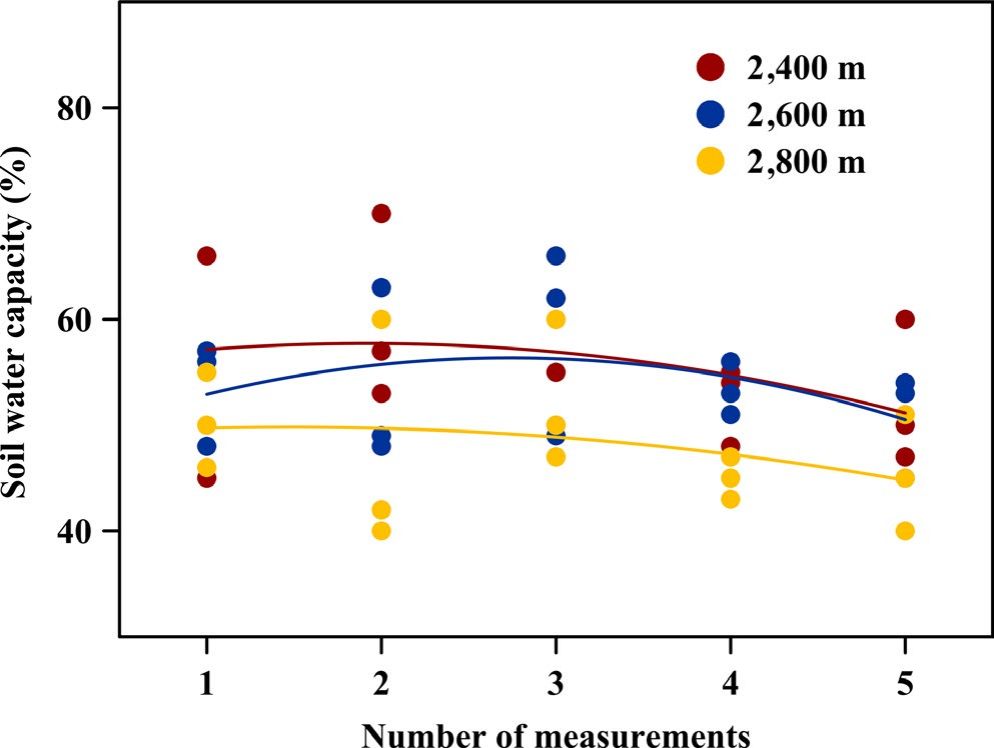
The soil water content of natural habitats at three altitudes in our field investigation. Solid red dots, solid blue dots, and solid yellow dots represent soil water capacity of 2,400 m, 2,600 m, and 2,800 m, respectively. Red line, blue line, and yellow line represent fitting curve (polynomial fitting) of soil water content at 2,400 m, 2,600 m, and 2,800 m, respectively. Five measurements of each field site were conducted at each altitude

In the middle of April 2016, we performed a field investigation on the sex ratio of *S. paraplesia* in the Wanglang National Nature Reserve (32°49′N–33°02′N, 103°55′E–104°10′E) along an altitude variation (2,400 m, 2,600 m and 2,800 m). This region (Figure 1) belongs to the monsoon climate zone with distinct dry (November to April) and wet (May to October) seasons. The mean annual precipitation ranged from 801 to 825 mm and changed a little among three altitudes (Peng, Wu, Xu, & Yang, 2012), and the air temperature in the growing season was 10–23°C. Based on United States Department of Agriculture Soil Taxonomy, the soil type at three altitudes was classified as Cryumbreps. We chose three plots (20 × 20 m) at each altitude in similar riparian environments (flat and similar water condition, Figure 2) and investigated all the male and female individuals according to their flowers. Each plot was >200 m interval. Multiple clones were considered as one individual if their roots were connected to each other. The sex ratio was determined as female to male (F/M), and chi‐square test was used to test significant departure from a 1:1 ratio.

### 2.2 Cuttings cultivated and drought treatment

**Figure 3:**
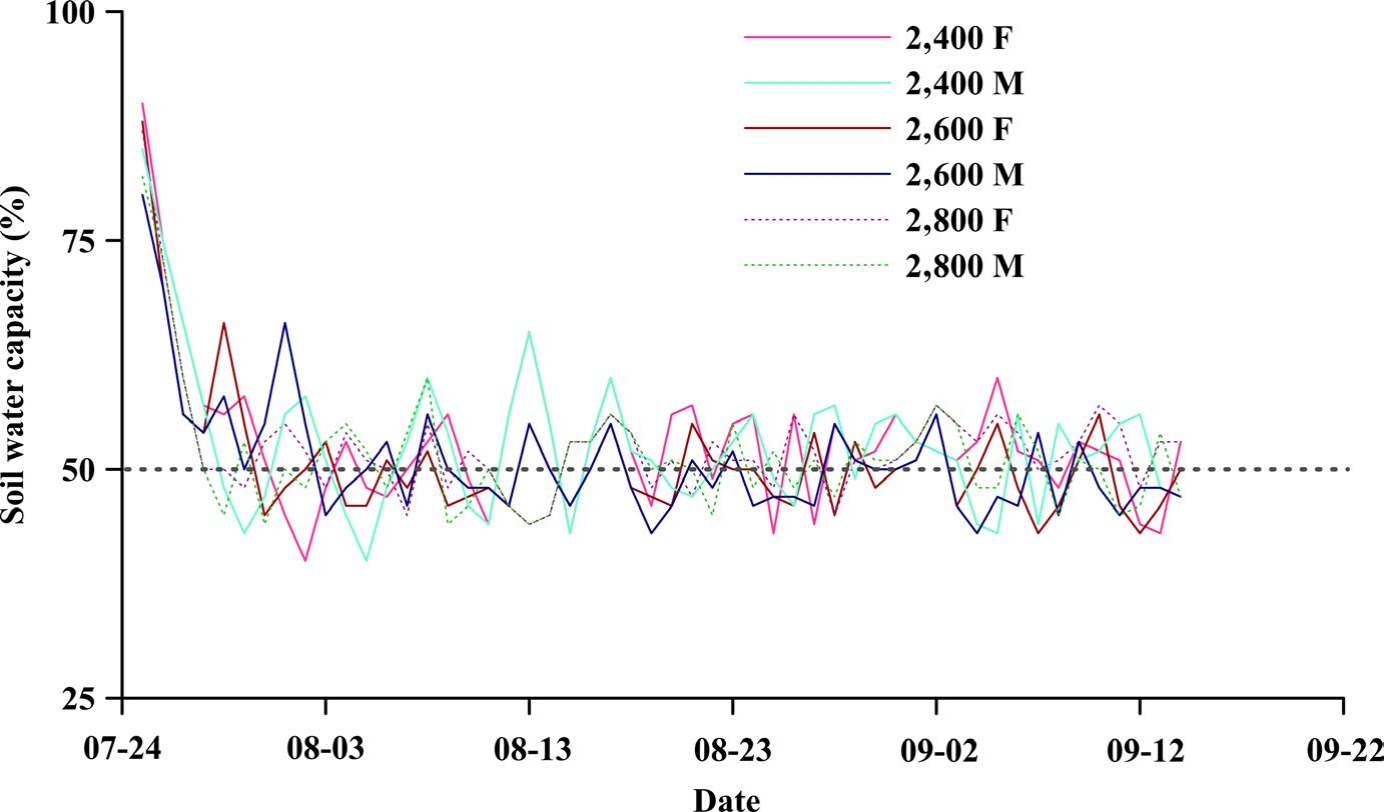
The soil water capacity characteristics of cuttings from three altitudes during drought treatment period. Turquoise, dark blue (solid line), and grass green (dotted line) lines represent males from altitudes of 2,400 m, 2,600 m, and 2,800 m, respectively. Hot pink, ruby red (solid line), and deep violet (dotted line) lines represent females from altitudes of 2,400 m, 2,600 m, and 2,800 m, respectively

In 2017, before flowering, the current‐year cuttings of *S. paraplesia* (approximately 15 cm) were collected from 10 different trees (5 males and 5 females) from 2,400 m, 2,600 m, and 2,800 m, respectively. All cuttings were planted into 10 L plastic pots filled with 8 kg of homogenized soil mixture (one cutting per pot). After naturally growing for approximately 3 months with optimal conditions in the Wanglang National Nature Reserve (2,600 m), 120 healthy cuttings with the similar growth and equal height were selected and transferred to growth chambers. Twelve growth chambers were installed at the altitude of 2,600 m in Wanglang National Nature Reserve. The growth chambers are nearly cylindrical structures with an automatic control system and equipment monitoring environmental elements (LT/WSK‐PLC; Copeland and Vaisala, Inc.). All the walls of the chambers were structured by transparent solar plates with 85% light transmittance. There are a total of 12 chambers, and four chambers were used in our study. The growth temperature was set to 10–27°C, and the relative humidity ranged from 50% to 80% according to the ambient conditions during July 25 to September 14, 2017, because the soil water capacity at three altitudes was ranged from 40% to 70% in the field investigation (Figure 2). We chose the 50% of soil water capacity as a moderate drought stress in this study. The experimental layout was a completely randomized design with 2 genders (male and female) × 3 altitudes (2,400 m, 2,600 m and 2,800 m) × 2 watering regimes (100% and 50% of soil water capacity). A moisture teller (TDR350; Spectrum Technologies Inc.) was used to measure the soil water every day (Figure 3). After the soil water declined to 50% of soil water capacity in each pot, they were kept constant by artificial water regulation. To prevent evaporation from the soil surface, the plastic bags were used to seal basal stems. The mean soil water capacity in each pot cuttings from three altitudes was ranged from 51% to 53% during the experimental period (Figure 3). Five cuttings of each treatment were selected, and the fourth fully expanded leaf of each cutting was collected and frozen in liquid nitrogen immediately. Next, they were stored at −80°C until further chemical analysis.

### 2.3 Growth and biomass measurements

The height was measured from the bottom to the top, and the basal diameter was determined on the shoot at 1 cm above the soil surface for all the cuttings. The height growth rate (GR_H_, cm day^−1^) and diameter growth rate (GR_D_, mm day^−1^) were calculated as follows: GR_H_ = (*H_t_*_2_ − *H_t_*_1_)/(*t*_2_ − *t*_1_) and GR_D_ = (*B_t_*_2_ − *B_t_*_1_)/(*t*_2_ − *t*_1_). *H_t_*_1_, *H_t_*_2_, *B_t_*_2_, and *B_t_*_1_ represented the height growth and basal diameter measured at the beginning (*t*_1_) and the end (*t*_2_) of the study, respectively. For the height growth and basal diameter measurement, fifteen replicates were used, and each measurement was recorded twice. To assess biomass, five cuttings from each sex and treatment were used for the biomass measurements at the end of the treatments. Cuttings were harvested and separated into leaves, stems and roots, and dried to a constant weight (70°C, 48 hr), followed by recording the leaf dry mass (LDM), stem dry mass (SDM), root dry mass (RDM), total dry mass (TDM), and calculated the aboveground mass/root mass ratio (AM/RM ratio). Each measurement was recorded twice.

### 2.4 Gas exchange and chlorophyll pigment measurements

Five replicates were conducted, and each measurement was recorded twice when determined the gas exchange and chlorophyll pigments. Gas exchange parameters were measured for the fourth or fifth fully expanded leaves from the apex. Five biological cuttings were randomly selected for each treatment and each sex. The net photosynthetic rate (*A*), transpiration rate (*E*), stomatal conductance (*g*_s_), intercellular CO_2_ concentration (*C*_i_), and ambient CO_2_ concentration (*C*_a_) were recorded at a saturating photosynthetic active radiation (1,500 μmol m^−2^ s^−1^) and a leaf temperature of 20°C using a portable photosynthesis system (LI‐6400XT; LI‐COR Inc.). As willow leaves are too slender to fully fill with the leaf chamber (2 × 3 cm), we marked each measured blade with a pencil along the outside of a standard leaf chamber and calculated their leaf area for *A*, *E,* and *C*_i_ corrections. The measured parameters were set to the following: leaf‐to‐air vapor pressure deficit was 1.5 ± 0.5 kPa, relative air humidity was 80%, and ambient CO_2_ concentration was 450 ± 5 μmol mol^−1^. Measurements were performed from 08:00 a.m. to 11:30 a.m. The ratio of *A* to *g_s_* was defined as the intrinsic water use efficiency (WUEi). The stomatal limitation value (SLV) was calculated according to Berry and Downton (1982) as the following formula: SLV = 1 − *C*_i_/*C*_a_.

Chlorophyll pigments were determined for the leaves used for gas exchange measurements. For chlorophyll pigment measurement, 80% (v/v) chilled acetone was used to soak leaf tissues (about 0.3 g) for approximately 20 hr under darkness until the leaf color was completely white. Next, the homogenates were centrifuged at 8,000 *g* for 10 min at 4°C. The supernatant was used to determine the absorbance of chlorophyll a (*Chl* a), chlorophyll b (*Chl* b), and carotenoids (*Caro*) at 663, 646, and 470 nm by a spectrophotometer (Unicam UV‐330; Unicam, Inc.). The sum of *Chl* a and *Chl* b was defined as total chlorophyll content (T*Chl*), according to Lichtenthaler (1987).

### 2.5 Leaf predawn water potential (*Ψ*_dawn_) and relative water content (RWC)

The fourth fully expanded leaves were used for *Ψ*_dawn _measurements by using a WP4 Dewpoint Potentiometer (WP4C; Decagon Devices, Inc.) before sunrise according to the operation manual. Leaf disks of five cuttings (avoiding the midrib) were randomly selected from each sex, each treatment to be fully fitted in a measure cup, and each measurement was recorded twice. After the measurement of *Ψ*_dawn_ and photosynthetic rate, leaf samples were collected to determine relative water content (RWC) (Weatherley, 1950). RWC = 100(FM − DM)/(TM − DM). Here, FM, DM, and TM represented fresh mass, turgid mass, and dry mass used for 10 leaf disks (0.8 cm diameter) from each leaf, respectively. And the same as the measurement of *Ψ*_dawn_, five replicates were conducted and each measurement was recorded twice.

### 2.6 Measurement of biochemical parameters

For each biochemical measurement, five replicates were used. To estimate the lipid peroxidation extent of the membrane, malondialdehyde (MDA) was measured according to the method described by Xu, Yang, et al. (2008). First, fresh leaves (about 0.3 g) were homogenized with 10 ml of 10% trichloroacetic acid and centrifuged at 12,000 *g* for 10 min. Next, 2 ml of 0.6% thiobarbituric acid with 10% trichloroacetic acid was added into 2 ml of the supernatants. Next, the mixture was boiled in a water bath for 15 min and cooled in an ice bath as soon as possible. The absorbance of the supernatants was measured at 450, 532, and 600 nm by using the spectrometer. The MDA content was calculated using the following formula: *C* (μM) = 6.45 (OD_532_ − OD_600_) − 0.56OD_450_. For relative electrolyte leakage (REL) measurement, after washed clearly, ten leaf disks were randomly collected from each biological replicate and soaked in small beakers with 10 ml of deionized water at room temperature for 6 hr. The electrical conductivity of the bathing solution was measured by a conductivity instrument (LC116; Mettler‐Toledo Instruments Co., Inc.) and recorded as C1. The beakers were subsequently incubated in a boiling water bath for 25 min and cooled to room temperature. The electrical conductivity was recorded as C2. REL = (C1/C2) × 100.

About 0.3 g of frozen leaf sample was ground in liquid nitrogen to a fine powder, and the peroxidase (POD) activity was measured by using 2 ml of 100 mM potassium phosphate buffer (pH 6.5) with 40 mM guaiacol, 10 mM H_2_O_2_, and an enzyme extract containing 100 μg of proteins at 25°C. The absorbance of the supernatants was determined at 470 nm (Chance & Maehly, 1955). The protein concentration was measured according to the Bradford method (Bradford, 1976).

Starch and fructose were extracted in a water bath with 80% (v/v) ethanol at 80°C for 30 min, followed by centrifugation at 8,000 *g* for 5 min. A total of 50 mg of dried fine powder from leaf samples was used. The supernatant was pooled into a 10 ml centrifuge tube, and 2 ml of 80% (v/v) ethanol was added. After being centrifuged at 5,000 *g* for 5 min, the supernatant was transferred into a new tube. Next, 80% (v/v) ethanol was added into the tube to a final volume of 8 ml. Starch and fructose were detected according to the methods of Dubois, Gilles, Hamilton, Rebers, and Smith (1956) and Murata, Akazawa, and Fukuchi (1968), respectively. Total soluble sugars were detected at 625 nm, following the method of Yemm and Willis (1954). Nonstructural carbohydrate (NSC) is defined as the sum of soluble sugars and starch.

### 2.7 Transmission electron microscopy observations

Two small sections (1–2 mm in length, avoiding the midrib) that picked out from the middle position of the fifth fully expanded leaves were used for transmission electron microscope (TEM) analysis. The leaf sections were fixed with 3% (v/v) glutaral pentanedial in 0.1 M phosphate buffer (pH 7.2) for 8 hr at 4°C and postfixed in 1% osmium tetroxide for 1 hr. Tissues were dehydrated in a graded ethanol series (50, 60, 70, 80, 90, 95, and 100%) and embedded in eponaraldite for 2 hr. Ultrathin sections (80 nm) were sliced, stained with uranyl acetate and lead citrate, and mounted on copper grids for observation via H‐600IV transmission electron microscopy (TEM; Hitachi, Inc.) (Zhang, Chen, Duan, Korpelainen, & Li, 2012).

### 2.8 Statistical analysis

To assess differences in all the measured parameters between the sexes, a multivariate analysis of variance (MANOVA) was performed. To estimate the effects of sex, altitude, watering, and their interaction, three‐way analysis of variance (ANOVAS) was performed. Individual differences among mean were determined by Duncan's test at the significance level of *p* ≤ 0.05. All the statistical analysis and principal component analysis (PCA) were conducted *via* the statistical software package for Windows (SPSS 18.0; SPSS Inc.). To show the variability of soil water capacity at each altitude in the field investigation, the fitting curves of soil water capacity were drawn according to the method of polynomial fitting using Golden Software Grapher 9 (Grapher 9; Golden Software Inc., LLC). Polynomial curve fitting was based on the equation as follows:

$$\text{Pn}\left(x\right)=A_{0}+A_{1}x+A_{2}x^{2}+A_{3}x^{3}+\text{…}+A_{n}x^{n}\;\left(n\leq N\right)$$

Pn(*x*) is a soil moisture polynomial fitted by the method of least square for experimental data (*x_i_*, *y_i_*) (*i* = 1, 2, 3…*N*). *x_i_*, *y_i_* represented numbers of measurement at each altitude and the soil water capacity of each measurement at each altitude, respectively. *N* represents the highest power of the polynomial. The value of *A*_0_, *A*_1_, *A*_2_…*A_n_* minimizes the sum of squares of deviations *F*(*A*_0_, *A_n_*) in the formula:

$$F\left(A_{0},A_{n}\right)=\sum_{\left(i=1\right)}^{n}{\left(A_{0}+A_{n}x_{i}-y_{i}\right)}^{2}$$

## 3 RESULTS

**Figure 4:**
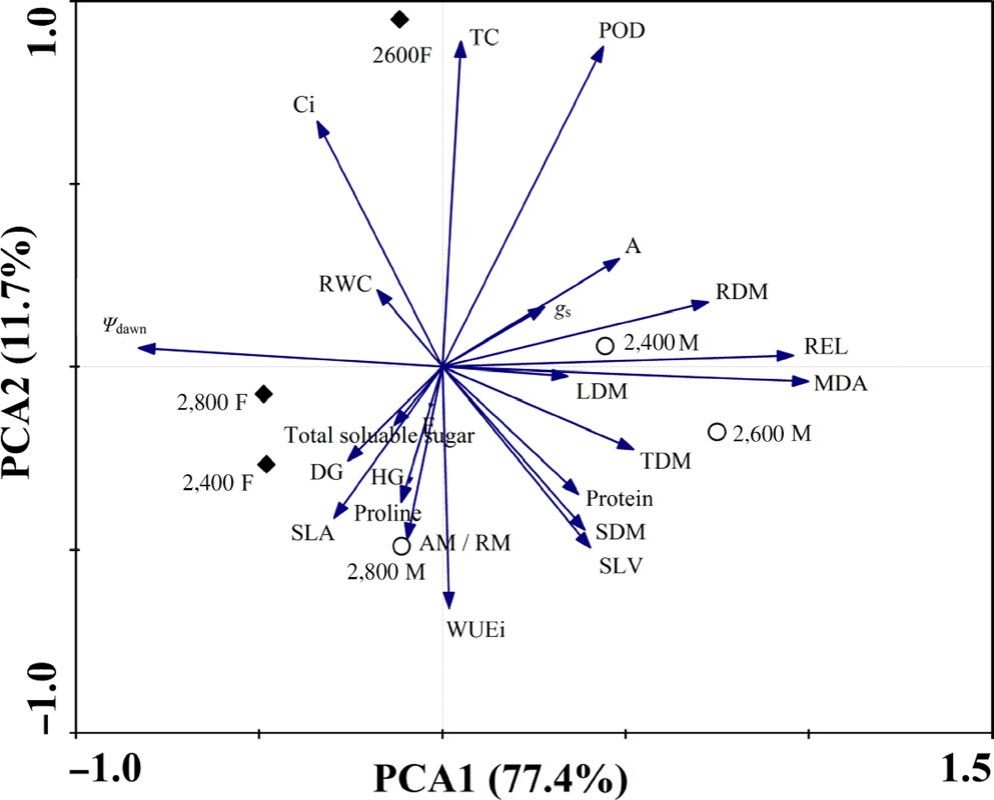
Principal component analysis analysis for all the indicators in leaves of male and female *Salix paraplesia* from three altitudes under drought‐stressed conditions (50% soil water capacity). Diamond represents females; circle represents males. 2400F, 2600F, and 2800F represent females from altitudes of 2,400 m, 2,600 m, and 2,800 m, respectively. 2400M, 2600M, and 2800M represent males from altitudes of 2,400 m, 2,600 m, and 2,800 m, respectively. PC1, principal component 1; PC2, principal component 2

As an overall assessment of the equality of mean vectors in several groups, MANOVA showed that the effects of sex, watering, altitude, and their interactions were significant at *p* ≤ 0.001 level (Table 1). Particularly, the *F* values were the highest for the effect of watering, which indicated that the watering effect was the most significant difference among all the factors. The PCA showed that the drought resistance index was rarely distributed in the area of females at 2,600 m under drought‐stressed conditions (Figure 4). However, a series of indicators were spread near to males at 2,400 m and 2,600 m, which were well‐separated from those at 2,800 m. Altogether, principal components 1 (PC1) and 2 (PC2) accounted for 77.4% and 11.7% of the observed variance, respectively.

**Table 1:** Results of multivariate analysis of variance (MANOVA) on all the morphological, physiological, and biochemical characteristics of *Salix paraplesia* males and females

Effect	Test statistic	Value	*F*	Hypothesis *df*	Error *df*	Significance
Sex	Pillai's Trace	0.987	83.207^a^	23.000	25.000	0.000
	Wilks' Lambda	0.013	83.207^a^	23.000	25.000	0.000
	Hotelling's Trace	76.551	83.207^a^	23.000	25.000	0.000
	Roy's Largest Root	76.551	83.207^a^	23.000	25.000	0.000
Watering effect	Pillai's Trace	0.999	1,059.634^a^	23.000	25.000	0.000
	Wilks' Lambda	0.001	1,059.634^a^	23.000	25.000	0.000
	Hotelling's Trace	974.863	1,059.634^a^	23.000	25.000	0.000
	Roy's Largest Root	974.863	1,059.634^a^	23.000	25.000	0.000
Altitude	Pillai's Trace	1.933	32.822	46.000	25.000	0.000
	Wilks' Lambda	0.000	63.859^a^	46.000	25.000	0.000
	Hotelling's Trace	235.733	122.991	46.000	25.000	0.000
	Roy's Largest Root	220.624	249.401^b^	23.000	25.000	0.000
Sex × Watering effect	Pillai's Trace	0.988	86.284^a^	23.000	52.000	0.000
	Wilks' Lambda	0.012	86.284^a^	23.000	50.000	0.000
	Hotelling's Trace	79.382	86.284^a^	23.000	48.000	0.000
	Roy's Largest Root	79.382	86.284^a^	23.000	26.000	0.000
Sex × Altitude	Pillai's Trace	1.932	31.908	46.000	25.000	0.000
	Wilks' Lambda	0.001	34.582^a^	46.000	25.000	0.000
	Hotelling's Trace	71.691	37.404	46.000	25.000	0.000
	Roy's Largest Root	52.600	59.461^b^	23.000	25.000	0.000
Watering effect × Altitude	Pillai's Trace	1.789	9.594	46.000	52.000	0.000
	Wilks' Lambda	0.001	29.779^a^	46.000	50.000	0.000
	Hotelling's Trace	167.993	87.649	46.000	48.000	0.000
	Roy's Largest Root	164.110	185.515^b^	23.000	26.000	0.000
Sex × Watering effect × Altitude	Pillai's Trace	1.747	7.816	46.000	52.000	0.000
	Wilks' Lambda	0.005	14.782^a^	46.000	50.000	0.000
	Hotelling's Trace	51.864	27.059	46.000	48.000	0.000
	Roy's Largest Root	48.563	54.898^b^	23.000	26.000	0.000

a Exact statistic.

b The statistic is upper bound of *F* that yields a lower bound for the significance level.

### 3.1 The sex ratio of field investigation

As shown in Table 2, a total of 253 willow trees (138 females and 115 males) were investigated in the Wanglang National Nature Reserve along an altitude variation with the soil water capacity from 40% to 70% (Figure 2). Fitting curve showed that soil water condition at 2,800 m was lower than 2,400 m and 2,600 m. The sex ratio of females to males (F/M) showed a tendency to increase with altitude, with 1.02, 1.27, and 1.40 at 2,400 m, 2,600 m, and 2,800 m, respectively. Although there was no significance for each study site, the total sex ratio of F/M was 1.20 (*p* = 0.025), showing a significant female bias.

**Table 2:** The investigation of sex ratio of female to male (F/M) *Salix paraplesia* along an altitude gradient (2,400, 2,600 and 2,800 m, respectively)

Altitude	Field sites	Number of females	Number of males	Ratio of F/M	*χ* ^2^	*p*
2,400 m	A1	16	13	1.23	6	0.199
	A2	19	20	0.95		
	A3	11	12	0.92		
	Total	46	45	1.02		
2,600 m	A4	33	21	1.57	8	0.092
	A5	7	9	0.77		
	A6	17	15	1.13		
	Total	57	45	1.27		
2,800 m	A7	13	9	1.44	8	0.092
	A8	15	10	1.50		
	A9	7	6	1.16		
	Total	35	25	1.40		
Total		138	115	1.20	88	0.025

Ratio of F/M was tested using a chi‐square test.

### 3.2 Growth and biomass accumulation

Overall, although the growth and biomass accumulation were not significantly affected by the interaction of sex × watering × altitude, the dry matter accumulation was significantly (*p* ≤ 0.05) affected by sex, drought and altitude (Table 3). Under control conditions, GR_H_, GR_D_, LDM, SDM, TDM, and AM/RM ratio were significantly higher in females than in male cuttings from 2,400 m. SDM, RDM, and TDM were significantly higher in females than in male cuttings from 2,600 m. However, the growth rate and dry matter accumulation exhibited little sexual difference in individuals at 2,800 m. Under drought‐stressed conditions, all the individuals exhibited lower values in growth and biomass accumulation. However, drought significantly decreased LDM, SDM, RDM, and TDM in female cuttings from 2,400 m, while it decreased LDM and RDM in male cuttings from 2,800 m.

**Table 3:** The morphological (growth, biomass accumulation, and distribution) indices of males and females *Salix paraplesia* under control (100% soil water capacity) and drought (50% soil water capacity) conditions at different altitudes

Watering	Altitude	Sex	GR_H_ (cm day^−1^)	GR_D_ (mm day^−1^)	LDM (g)	SDM (g)	RDM (g)	TDM (g)	AM/RM ratio
Control	2,400 m	Female	0.30 ± 0.03 ab	0.03 ± 0.00 a	0.95 ± 0.10 b	0.88 ± 0.08 bc	0.62 ± 0.08 ab	2.45 ± 0.22 b	3.04 ± 0.33 bc
		Male	0.19 ± 0.03 cd	0.02 ± 0.00 bc	0.60 ± 0.04 cd	0.42 ± 0.02 d	0.56 ± 0.04 bc	1.58 ± 0.06 c	1.84 ± 0.18 d
	2,600 m	Female	0.38 ± 0.07 a	0.03 ± 0.00 a	1.17 ± 0.14 a	1.26 ± 0.27 a	0.75 ± 0.06 a	3.18 ± 0.43 a	3.25 ± 0.43 ab
		Male	0.40 ± 0.07 a	0.03 ± 0.00 ab	1.03 ± 0.12 ab	0.96 ± 0.12 b	0.53 ± 0.09 bc	2.52 ± 0.28 b	4.14 ± 0.71 a
	2,800 m	Female	0.18 ± 0.02 cd	0.02 ± 0.00 cd	0.53 ± 0.02 cd	0.60 ± 0.09 cd	0.49 ± 0.03 bcd	1.63 ± 0.10 c	2.31 ± 0.13 bcd
		Male	0.12 ± 0.02 cd	0.02 ± 0.00 bc	0.51 ± 0.02 cd	0.38 ± 0.04 d	0.44 ± 0.05 cd	1.33 ± 0.07 cd	2.1 ± 0.21 cd
Drought	2,400 m	Female	0.17 ± 0.01 cd	0.01 ± 0.00 de	0.50 ± 0.07 cd	0.27 ± 0.04 d	0.34 ± 0.06 de	1.11 ± 0.15 cd	2.44 ± 0.30 bcd
		Male	0.16 ± 0.03 cd	0.01 ± 0.00 e	0.51 ± 0.05 cd	0.37 ± 0.04 d	0.42 ± 0.06 cd	1.30 ± 0.11 cd	2.28 ± 0.41 bcd
	2,600 m	Female	0.22 ± 0.02 bc	0.01 ± 0.00 de	0.63 ± 0.06 c	0.40 ± 0.05 d	0.49 ± 0.03 bcd	1.52 ± 0.12 c	2.12 ± 0.20 cd
		Male	0.19 ± 0.02 cd	0.01 ± 0.00 cde	0.66 ± 0.08 c	0.60 ± 0.07 cd	0.47 ± 0.05 bcd	1.73 ± 0.17 c	2.77 ± 0.32 cd
	2,800 m	Female	0.15 ± 0.02 cd	0.01 ± 0.00 cde	0.40 ± 0.03 de	0.44 ± 0.08 d	0.35 ± 0.05 de	1.18 ± 0.14 cd	2.47 ± 0.24 bcd
		Male	0.10 ± 0.02 d	0.01 ± 0.00 cde	0.27 ± 0.03 e	0.29 ± 0.07 d	0.22 ± 0.04 e	0.78 ± 0.13 d	2.54 ± 0.17 bcd
		*p* > *F*_S_	0.056	0.083	0.021	0.025	0.044	0.009	0.955
		*p* > *F*_W_	0.000	0.000	0.000	0.000	0.000	0.000	0.086
		*p* > *F*_A_	0.000	0.036	0.000	0.000	0.000	0.000	0.007
		*p* > *F*_S × W_	0.585	0.151	0.099	0.003	0.172	0.009	0.361
		*p* > *F*_S × A_	0.563	0.088	0.482	0.553	0.245	0.864	0.015
		*p* > *F*_W × A_	0.005	0.000	0.045	0.006	0.796	0.035	0.006
		*p* > *F*_S × W × A_	0.278	0.013	0.084	0.199	0.214	0.083	0.415

Note

Each value represents the mean ± *SE* (*n* = 5). GR_H_ and GR_D_ represent the mean ± *SE* (*n* = 15). Values followed by a different letter in the same column denote statistically significant differences between treatments at *p* ≤ 0.05 level according to Duncan's test.

Abbreviation(s): AM/RM ratio: aboveground mass/root mass ratio; *F*_A_: altitude effect; *F*_S_: sex effect; *F*_S × A_: interaction effect of sex and altitude; *F*_S × W_: interaction effect of sex and watering; *F*_S × W × A_: interaction effect of sex: watering and altitude; *F*_W_: watering effect; *F*_W × A_: interaction effect of watering and altitude; GR_D_: basal diameter growth rate; GR_H_: height growth rate; LDM: leaf dry mass; RDM: root dry mass; SDM: stem dry mass; TDM: total dry mass.

### 3.3 Gas exchange, chlorophyll pigment contents and leaf water relations

**Figure 5:**
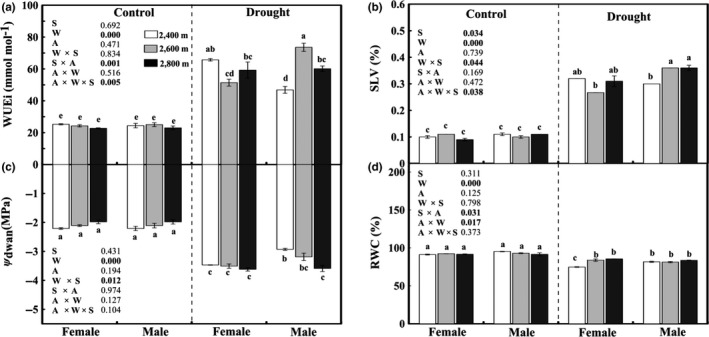
Intrinsic water use efficiency (WUEi; a), stomatal limitation value (SLV; b), leaf water potential (*ψ*_dawn_; c), and relative water content (RWC; d) in male and female *Salix paraplesia* under control (100% soil water capacity) and drought‐stressed (50% soil water capacity) conditions. The white bars, gray bars, and black bars represent altitudes of 2,400 m, 2,600 m, and 2,800 m, respectively. Bars represent the mean ± *SE* (*n* = 5). Bars with different letters are significantly different within the same panel (Duncan's test, *p* ≤ 0.05). S, sex effect; W, watering effect; A, altitude effect; W × S, interaction effect of sex and watering; S × A, interaction effect of sex and altitude; A × W, interaction effect of watering and altitude; A × W × S, interaction effect of sex, watering and altitude

Under control conditions, there was little sexual difference in gas exchange and chlorophyll pigments except for *A* and *g*_s _in cuttings from 2,600 m. The parameters of *C*_i_, *E*, *Chl a,* and* TChl* were significantly affected by the interaction of sex × watering × altitude (Table 4). Drought significantly decreased photosynthesis but increased *Chl a* and *TChl* contents in both male and female individuals. Compared between sexes, drought‐stressed males had significantly higher *E* than drought‐stressed females at 2,400 m. For individuals from 2,600 m and 2,800 m, drought‐stressed males had lower *C*_i_, *Chl a,* and* TChl* contents than drought‐stressed females. As shown in Figure 5, there was no sexual variation in WUEi, *ψ*_dawn_, and RWC under control conditions. Drought significantly increased WUEi value but decreased values of *ψ*_dawn_ and RWC in both sexes of three altitudes. Additionally, the lowest and the highest values of RWC and *ψ*_dawn _were in drought‐stressed females and males from 2,400 m, respectively.

**Table 4:** The net photosynthetic rate (*A*), stomatal conductance (*g*_s_), intercellular CO_2_ concentration (*C*_i_), transpiration rate (*E*), and chlorophyll pigment contents in *Salix paraplesia* males and females under control (100% soil water capacity) and drought‐stressed (50% soil water capacity) conditions from three altitudes

Watering	Altitude	Sex	*A* (μmol m^−2^ s^−1^)	*g_s_* (mol m^−2^ s^−1^)	*C*_i _(μmol mol^−1^)	*E* (m mol m^−2^ s^−1^)	*Chl a* (μg g^−1^ FW)	*Chl b* (μg g^−1^ FW)	*Caro* (μg g^−1^ FW)	*TChl* (μg g^−1^ FW)
Control	2,400 m	Female	16.17 ± 1.02 b	0.64 ± 0.02 bc	340.50 ± 3.73 a	4.24 ± 0.17 bc	779.08 ± 65.18 def	271.70 ± 20.11 de	175.23 ± 10.61 abc	1,051.00 ± 85.08 efg
		Male	13.37 ± 0.76 bc	0.57 ± 0.05 c	338.20 ± 4.51 a	3.52 ± 0.05 cde	704.13 ± 11.15 ef	264.41 ± 41.63 de	138.08 ± 5.60 d	968.54 ± 52.30 fg
	2,600 m	Female	19.42 ± 0.62 a	0.80 ± 0.03 a	337.39 ± 0.57 a	6.30 ± 0.47 a	683.33 ± 104.72 f	212.85 ± 43.01 e	176.31 ± 2.99 abc	896.18 ± 147.72 g
		Male	15.86 ± 0.58 b	0.63 ± 0.08 bc	343.58 ± 5.25 a	6.12 ± 0.28 a	721.12 ± 62.15 ef	229.51 ± 24.6 de	148.85 ± 2.58 cd	950.64 ± 86.69 fg
	2,800 m	Female	15.69 ± 0.64 b	0.69 ± 0.05 b	344.58 ± 1.06 a	4.40 ± 0.11 b	858.77 ± 29.85 cde	282.17 ± 30.55 de	160.29 ± 4.15 bcd	1,140.93 ± 51.47 cde
		Male	14.85 ± 1.52 b	0.65 ± 0.01 bc	339.37 ± 1.06 a	3.81 ± 0.09 bcd	844.19 ± 36.64 cde	293.11 ± 29.47 cde	160.97 ± 5.71 bcd	1,137.31 ± 65.38 cde
Drought	2,400 m	Female	7.08 ± 0.18 f	0.11 ± 0.00 e	259.46 ± 1.68 bc	1.90 ± 0.07 g	962.85 ± 19.16 abc	377.98 ± 8.22 abc	184.68 ± 11.93 ab	1,340.83 ± 27.28 abc
		Male	8.25 ± 0.45 ef	0.18 ± 0.02 de	265.77 ± 1.72 b	2.76 ± 0.23 ef	1,033.59 ± 8.86 ab	422.61 ± 15.74 a	185.74 ± 8.37 ab	1,456.21 ± 23.44 a
	2,600 m	Female	11.75 ± 2.17 cd	0.18 ± 0.02 d	265.77 ± 1.72 b	2.76 ± 0.23 de	1,038.45 ± 25.95 ab	397.65 ± 23.93 ab	205.44 ± 1.53 a	1,436.10 ± 47.96 ab
		Male	10.56 ± 0.29 de	0.15 ± 0.01 de	243.11 ± 8.41 c	2.31 ± 0.13 fg	863.33 ± 64.68 cde	283.08 ± 24.55 de	193.10 ± 12.18 ab	1,146.41 ± 86.96 cde
	2,800 m	Female	10.51 ± 0.35 de	0.21 ± 0.05 de	263.42 ± 16.42 b	2.87 ± 0.50 ef	1,075.41 ± 24.25 a	398.70 ± 40.96 ab	169.07 ± 22.2 bcd	1,474.11 ± 61.66 a
		Male	7.88 ± 0.21 ef	0.13 ± 0.00 de	243.12 ± 7.87 c	2.12 ± 0.09 fg	912.05 ± 33.2 bcd	315.20 ± 9.79 bcd	174.12 ± 12.51 abc	1,227.25 ± 41.93 bcd
		*p* > *F*_S_	0.003	0.005	0.034	0.019	0.070	0.188	0.056	0.084
		*p* > *F*_W_	0.000	0.000	0.000	0.000	0.000	0.000	0.000	0.000
		*p* > *F*_A_	0.000	0.015	0.739	0.000	0.034	0.034	0.128	0.037
		*p* > *F*_S × W_	0.161	0.134	0.044	0.502	0.213	0.090	0.112	0.134
		*p* > *F*_S × A_	0.491	0.054	0.169	0.134	0.432	0.222	0.229	0.315
		*p* > *F*_W × A_	0.733	0.406	0.427	0.000	0.201	0.273	0.204	0.189
		*p* > *F*_S × W × A_	0.086	0.269	0.038	0.012	0.035	0.074	0.490	0.033

Note

Each value is represented as the mean ± *SE* (*n* = 5). Values followed by a different letter in the same column denote statistically significant differences between treatments at *p* ≤ 0.05 level according to Duncan's test.

Abbreviation(s): *F*_A_: altitude effect; *F*_S × A_: interaction effect of sex and altitude; *F*_S × W_: interaction effect of sex and watering; *F*_S × W × A_: interaction effect of sex: watering and altitude; *F*_S_: sex effect; *F*_W × A_: interaction effect of watering and altitude; *F*_W_: watering effect.

### 3.4 Soluble protein, peroxidase activity, relative electrolyte leakage, and malondialdehyde

**Figure 6:**
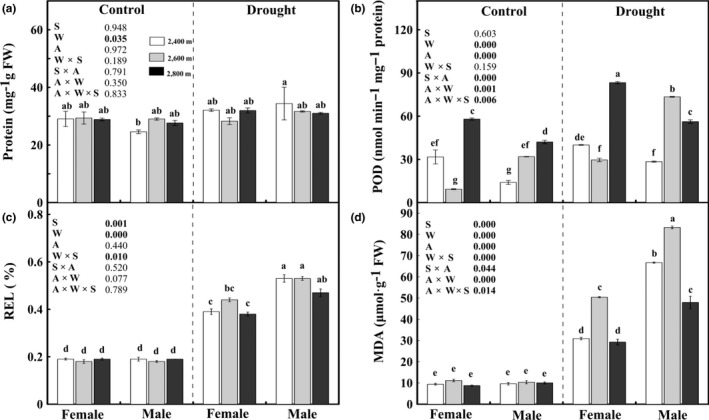
Protein content (Protein; a), peroxidase activity (POD; b), relative electric conductivity (REL; c), and malondialdehyde content (MDA; d) in the leaves of male and female *Salix paraplesia* under control (100% soil water capacity) and drought‐stressed (50% soil water capacity) conditions. The white bars, gray bars, and black bars represent altitudes of 2,400 m, 2,600 m, and 2,800 m, respectively. Bars represent the mean ± *SE* (*n* = 5). Bars with different letters are significantly different within the same panel (Duncan's test, *p* ≤ 0.05). S, sex effect; W, watering effect; A, altitude effect; W × S, interaction effect of sex and watering; S × A, interaction effect of sex and altitude; A × W, interaction effect of watering, and altitude; A × W × S, interaction effect of sex, watering, and altitude

Under control conditions, no sexual difference was found in the contents of total soluble protein and MDA or REL value (Figure 6). However, females exhibited a higher POD activity than males for cuttings from 2,400 m and 2,800 m but a lower activity for cuttings from 2,600 m. Drought significantly increased the values of POD, REL, and MDA in both sexes. Drought‐stressed females exhibited significantly lower REL, MDA content, and higher POD activity than drought‐stressed males, except for the POD activity in cuttings from 2,600 m. Additionally, POD and MDA were significantly affected by the interaction of sex × watering × altitude.

### 3.5 Contents of nonstructural carbohydrates

**Figure 7:**
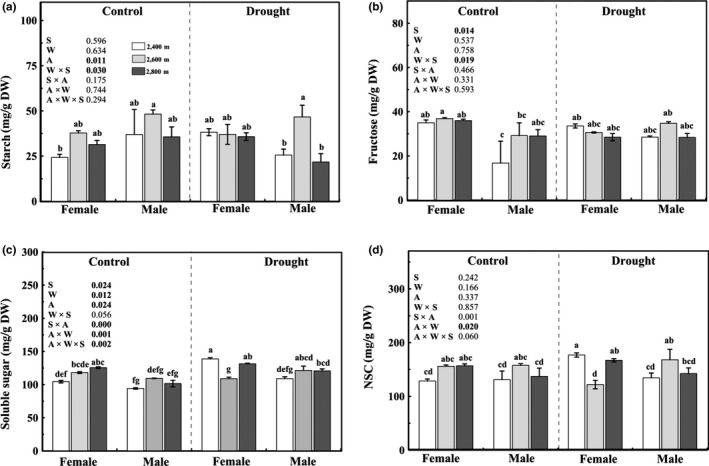
The contents of starch (Starch; a), fructose (Fructose; b), soluble sugar (Soluble sugar; c), and nonstructural carbohydrate (NSC; d) in the leaves of male and female *Salix paraplesia* under control (100% soil water capacity) and drought‐stressed (50% soil water capacity) conditions from three altitudes. Different letters above bars denote statistically significant differences at *p* ≤ 0.05 according to Duncan's test. Values are expressed as the mean ± *SE* (*n* = 5). W, watering condition effect; A, altitude effect; W × S, interaction effect of sex and watering condition; S × A, interaction effect of sex and altitude; A × W, interaction effect of watering condition and altitude; A × W × S, interaction effect of sex, watering condition, and altitude

Under control conditions, no sexual difference was detected in the content of starch or NSC in the leaves (Figure 7) of cuttings from all altitudes. However, the relatively higher contents of soluble sugar and fructose were found in female leaves. Under drought‐stressed conditions, female individuals from 2,400 m exhibited higher contents of soluble sugar and NSC than that of males. However, there was little sexual difference in starch content for cuttings from 2,400 m or 2,800 m. Additionally, only soluble sugar was significantly affected by the interaction of sex × watering × altitude.

### 3.6 Organella ultrastructure observation

**Figure 8:**
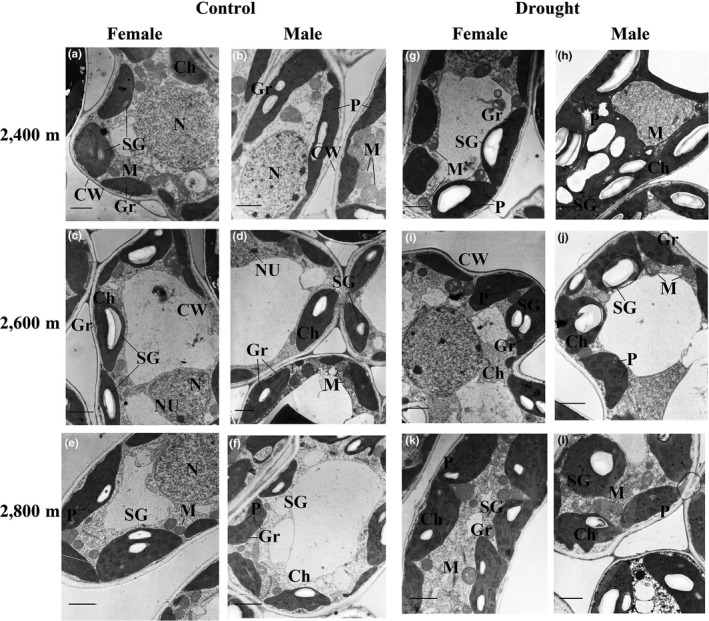
TEM observations of mesophyll cells in male and female *Salix paraplesia* under control (100% soil water capacity) and drought‐stressed (50% soil water capacity) conditions at different altitudes. (a), (c), (e) and (b), (d), (f) represent females and males at altitudes of 2,400 m, 2,600 m, and 2,800 m under control conditions, respectively; (g), (i), (k) and (h), (j), (l) represent females and males at altitudes of 2,400 m, 2,600 m, and 2,800 m under drought‐stressed conditions, respectively. The bars shown represent 1 μm. Ch: chloroplast; CW: cell wall; Gr: granum; N: nucleus; NU: nucleus; M: mitochondrion; P: plastoglobulus; SG: starch granule

Under control conditions, both sexes of *S. paraplesia* possessed smooth, clean, and well‐arranged thylakoid membranes in leaves (Figure 8a–f). The chloroplasts showed a lenticular shape, and the mitochondria exhibited a typical structure with clear cristae. Drought induced severe negative effects on their ultrastructure in both sexes, in which chloroplasts were changed into subcircular shape and the outline tended toward a wavy appearance (Figure 8g–l). Interestingly, the accumulation of starch granules in chloroplasts was observed in drought‐stressed males but was relatively lower in drought‐stressed females from three altitudes (Figure 8h,j,l). Although drought greatly increased the plastoglobule number in both sexes, drought‐stressed males exhibited more plastoglobules than drought‐stressed females, particularly for cuttings from 2,800 m.

## 4 DISCUSSION

Our field investigation on the adult trees showed that *S. paraplesia* was a female‐biased species in their natural population, particularly at a high altitude. This result is consistent with the species of *S. magnifica* along an altitude gradient in the Gongga Mountain, which is located in the eastern edge of the Tibetan Plateau (Lei, Chen, Jiang, Yu, & Duan, 2016). A previous study reported that the sex ratio of *Valeriana edulis* population was strongly female‐biased and altered across the elevation gradient (Petry et al., 2016). With respect to *Salix*, several studies have suggested the dominance of females due to their superior competitive ability and superior tolerance to environment disturbances (Hughes et al., 2010; Rottenberg, 2010).

According to empirical studies, reproduction would compete with vegetation growth and defense when resources are limited (Herms & Mattson, 1992). Despite the greater reproductive cost for females, the sex ratio of *Salix* populations was usually female‐biased in their harsh habitats (Lei et al., 2016; Ueno et al., 2007). One hypothesis is that females not only allocate more carbohydrates for reproduction but also usually invest more in defense for abiotic stress only if there is a demand. For example, high UV‐B radiation also induced females of *S. myrsinifolia* to be more tolerant than males because of their higher chlorogenic acid concentration (Nybakken, Hörkkä, & Julkunen‐Tiitto, 2012). If females invested more materials for defense than males, they could be more tolerant to drought stress, and this sex‐related tolerance could be possibly exhibited in the tree vegetative growth stage. In our study, we found that the cuttings of *S. paraplesia* females from 2,800 m possessed a higher self‐protective ability for their higher antioxidant enzyme activities (Reddy, Chaitanya, & Vivekanandan, 2004), less lipid peroxidation, and cellular membrane damage than males under drought stress, as reflected by their higher POD activity, lower MDA content, and REL. Under drought‐stressed conditions, the accumulation of reactive oxygen species (ROS) can induce oxidative stress on plant cells and disorder of cellular structures (Zhang et al., 2012), while the higher POD activity can efficiently eliminate the massive amount of H_2_O_2_ and protect the integrity and functionality of cellular membranes and other organelles. Individuals have a higher photosynthetic capacity, greater accumulation of substances for osmotic adjustment, and a more efficient enzymatic detoxification cycle are considered to have a better protective abilities. Therefore, *S. paraplesia* females avoid negative effects induced by drought stress for their better self‐protective ability. Alternatively, the reason why our results differed from previously postulated studies that males are more physiologically plastic with respect to water use (Dudley, 2006; Dudley & Galen, 2007) was perhaps due to the different water stress intensity, developmental stage, and water‐use efficiency among of species. We also predicted that male and female seedlings in this period only make vegetative investments instead of reproductive efforts (Juvany & Munné‐Bosch, 2015; Sánchez‐Vilas, Bermúdez, & Retuerto, 2012). Second, varied sex‐related physiological performances under drought stress may also be related to high genetic divergence of willow trees.

Net photosynthetic rate and stomatal conductance are indicators of drought tolerance. Drought stress significantly reduces the net photosynthetic rate, but the drought‐resistant species showed less decrease (Han, Luo, Li, Korpelainen, & Li, 2018; Xu, Peng, et al., 2008; Xu, Yang, et al., 2008; Zhang et al., 2010, 2014). In our study, under well‐watered conditions, *S. paraplesia* females did not display a significantly higher photosynthetic capacity than males to compensate the higher cost of reproduction in females (Obeso, 2002). However, *S. paraplesia* females exhibited a higher *TChl* content and net photosynthetic rate under drought‐stressed conditions for the high altitude, which resulted in females outperforming males in carbon assimilation. Carbon assimilation is important modulator of plant acclimation to environmental stress. More starch, soluble sugar, and NSC suggested *S. paraplesia* females (low and high altitude) were more tolerant to environmental stress than males. Female cuttings from 2800m had higher TDM, net photosynthetic rate, stomatal conductance, and NSC content than that of males, but theie difference was no statistical significance perhaps because of the high variance among clones. Under drought conditions, the stomata were closed, transpiration decreased, and the water and nutrient flow diminished due to a reduced diffusion rate of nutrients in the soil to the absorbing root surface. Therefore, nutritional imbalance depressed plant growth (Pinkerton & Simpson, 1986; Rouphael, Cardarelli, Schwarz, Franken, & Colla, 2012). Our results were consistent with a previous report that *S. paraplesia* females could maintain a faster growth ratio for survival under nutrient limitations than males (Jiang et al., 2016). Additionally, for other dioecious species, females showed a more conservative strategy in water use, with a higher photosynthetic rate than males to achieve a better tolerance (Álvarez‐Cansino et al., 2010; Rakocevic et al., 2009). Therefore, our results support the hypothesis that *S. paraplesia* female cuttings from 2,800 m showed better drought tolerance than males. However, *S. paraplesia* cuttings from 2,400 m and 2,600 m did not exhibit obvious sex‐biased responses to drought stress.

There are also sex‐related variations with altitude and response to drought stress. An increased underground investment in plants could enhance their stress resistance (Huan, Wang, Liu, Xu, & He, 2016; Kano, Inukai, Kitano, & Yamauchi, 2011; Ma et al., 2015). Under drought‐stressed conditions, larger root system and total biomass accumulation combined with lower REL and MDA content were detected in females than in males from high altitude but not in those from low altitude under drought‐stressed conditions. This result suggested that the growth of *S. paraplesia* females was favored at a high altitude and that there was a female‐biased pattern at a high altitude. Additionally, the slightly female‐biased response of NSC together with higher POD activity in *S. paraplesia* females from high altitude further reflected that females from high altitude possessed a greater self‐protective ability than that of males. These sex‐related variations to drought stress may be because of the difference in their constitutive defense costs, in which females will produce more expensive compounds only when it is necessary (Lei et al., 2017; Nybakken et al., 2012). Furthermore, *S. paraplesia* cuttings that originally grew in the middle altitude exhibited the best growth under control and drought‐stressed conditions. This result could explain the distribution of *S. paraplesia*, which ranged from 2,200 m to 3,000 m, mainly at approximately 2,600 m in the Wanglang National Nature Reserve. According to our data, a marked difference was observed in the MDA content, POD activity between the high altitude and low altitude, which showed that drought stress limited more *S. paraplesia* cuttings from low altitude enzymatic detoxification than high altitude. This could be interpreted as willows at high altitude may be more adaptable to stress environment for they are exposed to harsher conditions (including extremely lower temperature, drier, and stronger ultraviolet radiation) for a long period. Males and females from different altitude might have different evolution speed with long‐term various environmental stresses, which lead to amplify or reduce the sexual difference and different sex‐related responses to stress environment (He et al., 2017; Huang et al., 2018). As to the reason why drought tolerance between sexes differed among different altitude, perhaps because the high variance between clones might musk the true sex‐related effects responding to drought stress.

In conclusion, our study provides some evidences that there are sexually differential responses to drought in *S. paraplesia*, and females are more tolerant than males, particularly for the high‐altitude individuals. It is reasonable to predict that *S. paraplesia* females probably will be superior to males when subjected to drought conditions in the alpine areas.

## CONFLICT OF INTEREST

None declared.

## AUTHOR CONTRIBUTION

JL, HS, and DT performed the experiments and collected the data. JL analyzed the data and wrote the manuscript. SZ designed the research project, supervised experiments, and improved the manuscript.

## DATA AVAILABILITY STATEMENT

All data used are presented in the manuscript.

## References

[ece35175-bib-0001] Álvarez‐Cansino, L. , Zunzunegui, M. , Díaz‐Barradas, M. C. , & Esquivias, M. P. (2010). Physiological performance and xylem water isotopic composition underlie gender‐specific responses in the dioecious shrub *Corema album* . Physiologia Plantarum, 140, 32–45. 10.1111/j.1399-3054.2010.01382.x 20487375

[ece35175-bib-0002] Berry, J. A. , & Downton, W. J. S. (1982). Environmental regulation of photosynthesis In Govindjee J. A. (Ed.), Photosynthesis vol II development. Carbon metabolism and plant productivity (pp. 267–273). London, UK: Academic Press.

[ece35175-bib-0003] Bradford, M. M. (1976). A rapid and sensitive method for the quantitation of microgram quantities of protein utilizing the principle of protein‐dye binding. Analytical Biochemistry, 72, 248–254. 10.1016/0003-2697(76)90527-3 942051

[ece35175-bib-0004] Chance, B. , & Maehly, A. C. (1955). Assay of catalase and peroxidases. Methods in Enzymology, 11, 764–775. 10.1016/S0076-6879(55)02300-8

[ece35175-bib-0005] Chen, J. , Duan, B. L. , Wang, M. L. , Korpelainen, H. , & Li, C. Y. (2014). Intra‐ and inter‐sexual competition of *Populus cathayana* under different watering regimes. Functional Ecology, 28, 124–136. 10.1111/1365-2435.12180

[ece35175-bib-0006] Dawson, T. E. , & Bliss, L. C. (1989). Patterns of water use and the tissue water relations in the dioecious shrub, *Salix arctica*: The physiological basis for habitat partitioning between the sexes. Oecologia, 79, 332–343. 10.1007/BF00384312 23921398

[ece35175-bib-0007] Dubois, M. , Gilles, K. A. , Hamilton, J. K. , Rebers, P. A. , & Smith, F. (1956). Colorimetric method of determination of sugars and related substances. Analytical Chemistry, 28, 350–356. 10.1021/ac60111a017

[ece35175-bib-0008] Dudley, L. (2006). Ecological correlates of secondary sexual dimorphism in *Salix glauca* (Salicaceae). American Journal of Botany, 93, 1775–1783. 10.3732/ajb.93.12.1775 21642123

[ece35175-bib-0009] Dudley, L. , & Galen, C. (2007). Stage‐dependent patterns of drought tolerance and gas exchange vary between sexes in the alpine willow, *Salix glauca* . Oecologia, 153, 1–9. 10.1007/s00442-007-0712-4 17406906

[ece35175-bib-0010] Fisher, R. (1930). The theory of natural selection. London, UK: Oxford University Press.

[ece35175-bib-0011] Han, Q. Q. , Luo, J. X. , Li, Z. J. , Korpelainen, H. , & Li, C. Y. (2018). Improved drought resistance by intergeneric graftingin Salicaceae plants under water deficits. Environmental and Experimental Botany, 155, 217–225. 10.1016/j.envexpbot.2018.07.003

[ece35175-bib-0012] He, J. D. , Dong, T. F. , Huang, K. C. , Yang, Y. X. , Li, D. D. , Xu, X. , & He, X. H. (2017). Sex‐specific floral morphology, biomass, and phytohormones associated with altitude in dioecious *Populus cathayana* populations. Ecology and Evolution, 7, 3976–3986. 10.1002/ece3.2808 28616192 PMC5468146

[ece35175-bib-0013] Herms, D. A. , & Mattson, W. J. (1992). The dilemma of plants: To grow or defend. The Quarterly Review of Biology, 67, 283–335. 10.1086/417659

[ece35175-bib-0014] Huan, H. H. , Wang, B. X. , Liu, G. , Xu, X. , & He, X. H. (2016). Sexual differences in morphology and aboveground biomass allocation in relation to branch number in *Morus alba* saplings. Australian Journal of Botany, 64, 269–275. 10.1071/BT15189

[ece35175-bib-0015] Huang, K. C. , Liao, Y. M. , Dong, T. F. , Yang, Y. X. , He, J. D. , Huan, H. H. , … Xu, X. (2018). Sex‐specific responses of tree‐ring growth to climate associated with altitude in the dioecious tree *Populus cathayana* . Journal of Plant Ecology, 11, 771–779. 10.1093/jpe/rtx048

[ece35175-bib-0016] Hughes, F. M. R. , Johansson, M. E. , Xiong, S. J. , Carlborg, E. , Hawkins, D. , Svedmark, M. , … Nilsson, C. (2010). The influence of hydrological regimes on sex ratios and spatial segregation of the sexes in two dioecious riparian shrub species in northern Sweden. Plant Ecology, 208, 77–92. 10.1007/s11258-009-9689-x

[ece35175-bib-0017] Jiang, H. , Zhang, S. , Lei, Y. B. , Xu, G. , & Zhang, D. (2016). Alternative growth and defensive strategies reveal potential and gender specific trade‐offs in dioecious plants *Salix paraplesia* to nutrient availability. Frontiers in Plant Science, 7, 1064 10.3389/fpls.2016.01064 27489556 PMC4951494

[ece35175-bib-0018] Juvany, M. , & Munné‐Bosch, S. (2015). Sex‐related differences in stress tolerance in dioecious plants: A critical appraisal in a physiological context. Journal of Experimental Botany, 66, 6083–6092. 10.1093/jxb/erv343 26163697

[ece35175-bib-0019] Kano, M. , Inukai, Y. , Kitano, H. , & Yamauchi, A. (2011). Root plasticity as the key root trait for adaptation to various intensities of drought stress in rice. Plant and Soil, 342, 117–128. 10.1007/s11104-010-0675-9

[ece35175-bib-0020] Lei, Y. B. , Chen, K. , Jiang, H. , Yu, L. , & Duan, B. L. (2016). Contrasting responses in the growth and energy utilization properties of sympatric *Populus* and *Salix* to different altitudes: Implications for sexual dimorphism in Salicaceae. Physiologia Plantarum, 159, 30–41. 10.1111/ppl.12479 27300648

[ece35175-bib-0021] Lei, Y. B. , Jiang, Y. L. , Chen, K. , Duan, B. L. , Zhang, S. , Korpelainen, H. , … Li, C. Y. (2017). Reproductive investments driven by sex and altitude in sympatric *Populus* and *Salix* trees. Tree Physiology, 37, 1503–1514. 10.1093/treephys/tpx075 28985430

[ece35175-bib-0022] Lichtenthaler, H. K. (1987). Chlorophyll and carotenoids: Pigments of photosynthetic biomembranes. Methods in Enzymology, 148, 350–382. 10.1016/0076-6879(87)48036-1

[ece35175-bib-0023] Ma, L. , Sun, X. D. , Kong, X. X. , Galvan, J. V. , Li, X. , Yang, S. H. , … Hu, X. Y. (2015). Physiological, biochemical and proteomics analysis reveals the adaptation strategies of the alpine plant *Potentilla saundersiana* at altitude gradient of the northwestern Tibetan plateau. Journal of Proteomics, 112, 63–82. 10.1016/j.jprot.2014.08.009 25181701

[ece35175-bib-0024] Murata, T. , Akazawa, T. , & Fukuchi, S. (1968). Enzymic mechanism of starch of starch breakdown in germinating rice seeds I. An analytical study. Plant Physiology, 43, 1899–1905. 10.1104/pp.43.12.1899 16656988 PMC1087101

[ece35175-bib-0025] Nybakken, L. , Hörkkä, R. , & Julkunen‐Tiitto, R. (2012). Combined enhancements of temperature and UVB influence growth and phenolics in clones of the sexually dimorphic *Salix myrsinifolia* . Physiologia Plantarum, 145, 551–564. 10.1111/j.1399-3054.2011.01565.x 22212028

[ece35175-bib-0026] Obeso, J. R. (2002). The costs of reproduction in plants. New Phytologist, 155, 320–348. 10.1046/j.1469-8137.2002.00477.x 33873312

[ece35175-bib-0027] Öztürk, M. , Hakeem, K. R. , Faridah‐Hanum, I. , & Efe, R. (2015). Climate change impacts on high‐altitude ecosystems. Switzerland: Springer International Publishing Switzerland 10.1007/978-3-319-12859-7

[ece35175-bib-0028] Peng, G. Q. , Wu, C. C. , Xu, X. , & Yang , D. M., (2012). The age‐related changes of leaf structure and biochemistry in juvenile and mature subalpine fir trees (*Abies faxoniana* Rehder & E.H. Wilson.) along an altitudinal gradient. Polish Journal of Ecology, 60, 311–321.

[ece35175-bib-0029] Petry, W. K. , Soule, J. D. , Iler, A. M. , Chicas‐Mosier, A. , Inouye, D. W. , Miller, T. E. , & Mooney, K. A. (2016). Sex‐specific responses to climate change in plants alter population sex ratio and performance. Science, 353, 69–71. 10.1126/science.aaf2588 27365446

[ece35175-bib-0030] Pinkerton, A. , & Simpson, J. R. (1986). Interactions of surface drying and subsurface nutrients affecting plant‐growth on acidic soil profiles from an old pasture. Australian Journal of Experimental Agriculture, 26, 681–689. 10.1071/EA9860681

[ece35175-bib-0031] Rakocevic, M. , Medrado, M. J. S. , Martim, S. F. , & Assad, E. D. (2009). Sexual dimorphism and seasonal changes of leaf gas exchange in the dioecious tree *Ilex paraguariensis* grown in two contrasted cultivation types. Annals of Applied Biology, 154, 291–301. 10.1111/j.1744-7348.2008.00298.x

[ece35175-bib-0032] Reddy, A. R. , Chaitanya, K. V. , & Vivekanandan, M. (2004). Drought‐induced responses of photosynthesis and antioxidant metabolism in higher plants. Journal of Plant Physiology, 161, 1189–1202. 10.1016/j.jplph.2004.01.013 15602811

[ece35175-bib-0033] Renner, S. S. (2014). The relative and absolute frequencies of angiosperm sexual systems: Dioecy, monoecy, gynodioecy, and an updated online database. American Journal of Botany, 101, 1588–1596. 10.3732/ajb.1400196 25326608

[ece35175-bib-0034] Renner, S. S. , & Ricklefs, R. E. (1995). Dioecy and its correlates in the flowering plants. American Journal of Botany, 82, 596–606. 10.1002/j.1537-2197.1995.tb11504.x

[ece35175-bib-0035] Rottenberg, A. (2010). Sex ratio and gender stability in the dioecious plants of Israel. Botanical Journal of the Linnean Society, 128, 137–148. 10.1111/j.1095-8339.1998.tb02112.x

[ece35175-bib-0036] Rouphael, Y. , Cardarelli, M. , Schwarz, D. , Franken, P. , & Colla, G. (2012). Effects of drought on nutrient uptake and assimilation in vegetable crops In Aroca R. (Ed.), Plant responses to drought stress (pp. 171–195). Berlin, Germany: Springer.

[ece35175-bib-0037] Sánchez‐Vilas, J. , Bermúdez, R. , & Retuerto, R. (2012). Soil water content and patterns of allocation to below‐ and above‐ground biomass in the sexes of the subdioecious plant *Honckenya peploides* . Annals of Botany, 110, 839–848. 10.1093/aob/mcs157 22782243 PMC3423814

[ece35175-bib-0038] Tognetti, R. (2012). Adaptation to climate change of dioecious plants: Does gender balance matter? Tree Physiology, 32, 1321–1324. 10.1093/treephys/tps105 23139060

[ece35175-bib-0039] Trenberth, K. E. , Dai, A. , Der Schrier, G. V. , Jones, P. D. , Barichivich, J. , Briffa, K. R. , & Sheffield, J. (2014). Global warming and changes in drought. Nature Climate Change, 4, 17–22. 10.1038/nclimate2067

[ece35175-bib-0040] Ueno, N. , Suyama, Y. , & Seiwa, K. (2007). What makes the sex ratio female‐biased in the dioecious tree *Salix sachalinensis*? Journal of Ecology, 95, 951–959. 10.1111/j.1365-2745.2007.01269.x

[ece35175-bib-0041] Vega‐Frutis, R. , Varga, S. , & Kytöviita, M. M. (2013). Dioecious species and arbuscular mycorrhizal symbioses: The case of *Antennaria dioica* . Plant Signaling and Behavior, 8, 3 10.4161/psb.23445 PMC3676512 23299337

[ece35175-bib-0042] Weatherley, P. E. (1950). Studies in the water relations of the cotton plant. I. The field measurement of water deficit in leaves. New Phytologist, 49, 81–97. 10.1111/j.1469-8137.1950.tb05146.x

[ece35175-bib-0043] Wu, Q. P. , Tang, Y. , Dong, T. F. , Liao, Y. M. , Li, D. D. , He, X. H. , & Xu, X. (2018). Additional AM fungi inoculation increase *Populus cathayana* intersexual competition. Frontiers in Plant Science, 9, 607 10.3389/fpls.2018.00607 29868065 PMC5951968

[ece35175-bib-0044] Xu, X. , Peng, G. Q. , Wu, C. C. , Korpelainen, H. , & Li, C. Y. (2008). Drought inhibits photosynthetic capacity more in females than in males of *Populus cathayana* . Tree Physiology, 28, 1751–1759. 10.1093/treephys/28.11.1751 18765380

[ece35175-bib-0045] Xu, X. , Yang, F. , Xiao, X. W. , Zhang, S. , Korpelainen, H. , & Li, C. Y. (2008). Sex‐specific responses of *Populus cathayana* to drought and elevated temperatures. Plant Cell & Environment, 31, 850–860. 10.1111/j.1365-3040.2008.01799.x 18284585

[ece35175-bib-0046] Yemm, E. W. , & Willis, A. J. (1954). The estimation of carbohydrates in plant extracts by anthrone. Biochemical Journal, 34, 508–514. 10.1042/bj0570508 PMC1269789 13181867

[ece35175-bib-0047] Zhang, S. , Chen, F. G. , Peng, S. M. , Ma, J. W. , Korpelainen, H. , & Li, C. Y. (2010). Comparative physiological, ultrastructural and proteomic analyses reveal sexual differences in the responses of *Populus cathayana* under drought stress. Proteomics, 10, 2661–2677. 10.1002/pmic.200900650 20455211

[ece35175-bib-0048] Zhang, S. , Chen, L. G. , Duan, B. L. , Korpelainen, H. , & Li, C. Y. (2012). *Populus cathayana* males exhibit more efficient protective mechanisms than females under drought stress. Forest Ecology and Management, 275, 68–78. 10.1016/j.foreco.2012.03.014

[ece35175-bib-0049] Zhang, S. , Jiang, H. , Zhao, H. X. , Korpelainen, H. , & Li, C. Y. (2014). Sexually different physiological responses of *Populus cathayana* to nitrogen and phosphorus deficiencies. Tree Physiology, 34, 343–354. 10.1093/treephys/tpu025 24739232

